# A Review of Four Practice Guidelines of Inflammatory Bowel Disease

**DOI:** 10.7759/cureus.16859

**Published:** 2021-08-03

**Authors:** Okelue E Okobi, Iboro O Udoete, Oyinlola O Fasehun, Tobechukwu Okobi, Endurance O Evbayekha, Joanna J Ekabua, Henry Elukeme, Imoh L Ebong, Olamide O Ajayi, Iyanu V Olateju, Anthonette Taiwo, Ifeoma C Anaya, Janet A Omole, Mireille B Nkongho, Ugochi Ojinnaka, Abimbola O Ajibowo, Omosefe E Ogbeifun, Osemwegie O Ugbo, Ovie Okorare, Zainab Akinsola, Rahman A Olusoji, Ijeoma O Amanze, Jane N Nwafor, Nnenna A Ukoha, Thomas A Elimihele

**Affiliations:** 1 Family Medicine, Lakeside Medical Center, Belle Glade, USA; 2 Public Health, Central Michigan University, Mount Pleasant, USA; 3 Internal Medicine, University College Hospital, Ibadan, NGA; 4 Internal Medicine, Bronx Care Health System, New York, USA; 5 Isolation/Internal Medicine, Stella Obasanjo Isolation Center, Benin, NGA; 6 Infectious Disease, University of Louisville, Louisville, USA; 7 Internal Medicine, Floyd Medical Center, Rome, USA; 8 Internal Medicine, University of Ghana School of Medicine and Dentistry, Accra, GHA; 9 Internal Medicine, Obafemi Awolowo College of Health Sciences, Olabisi Onabanjo University, Sagamu, NGA; 10 Internal Medicine, Washington Adventist University, Takoma Park, USA; 11 Internal Medicine/Health Information Management, Betsy Johnson Hospital, Dunn, USA; 12 Pathology and Laboratory Medicine, Ahmadu Bello University, Zaria, NGA; 13 Internal Medicine, California Institute of Behavioral Neurosciences and Psychology, Fairfield, USA; 14 Psychiatry, Saint James School of Medicine, Saint Vincent, VCT; 15 Family Medicine, Lankenau Medical Center, Wynnewood, USA; 16 Internal Medicine, Lugansk Medical University, Lugansk, UKR; 17 Public Health, University of West Florida, Florida, USA; 18 Oncology Research, Baylor Scott & White Health, Dallas, USA; 19 Internal Medicine, Delta State University, Abraka, NGA; 20 Internal Medicine, Windsor University School of Medicine, Toronto, CAN; 21 Internal Medicine, St. Helens and Knowsley Teaching Hospitals National Health Service, Prescot, GBR; 22 Internal Medicine, Molly Specialist Hospital, Ibadan, NGA; 23 Internal Medicine, University of the District of Columbia, Silver Spring, USA; 24 Internal Medicine, Royal Cross Methodist Hospital, Abia, NGA; 25 Clinical Research, Norris Comprehensive Cancer Center, University of Southern California, Los Angeles, USA

**Keywords:** ulcerative colitis (uc), guideline, treatment choices, inflammatory bowel disease, crohn's disease

## Abstract

Inflammatory bowel disease (IBD) is a term that encompasses conditions characterized by chronic inflammation of the gastrointestinal tract (GIT). It includes Crohn's disease and ulcerative colitis. Major scientific organizations interested in gastrointestinal systems or GIT-focused organizations worldwide release guidelines for diagnosing, classifying, managing, and treating IBD. However, there are subtle differences among each of these guidelines. This review evaluates four evidence-based guidelines in the management of IBD and seeks to highlight the differences and similarities between them. The main differences in the evaluated guidelines were in diagnosis and treatment recommendations. The diagnosing recommendations were comparable amongst the four guidelines; however, some were more specific about limiting the number of interventions necessary to confirm a diagnosis. Regarding treatment options, each guideline had clear suggestions about what was considered ideal. Although the treatment options were identical, the main differences existed in the recommended diets and initial therapy in patients with moderate disease. Clinical practice guidelines (CPGs) recommend evidence-based practice from opinion leaders in clinical decision-making. Rather than dictating a one-size-fits-all approach in IBD management, reviewing various guidelines can enhance the cross-pollination of ideas amongst clinicians to improve decision-making. Clearly describing and appraising evidence-based reasoning for scientific recommendations remain driving factors for quality patient care. The effectiveness of CPGs in improving health and the complexities of their formation requires constant review to maximize constructive criticisms and explore possible improvements.

## Introduction and background

Inflammatory bowel disease (IBD) is a broad term that includes chronic remittent inflammatory conditions of the gastrointestinal tract - Crohn's disease (CD) and ulcerative colitis (UC). They are both clinically diverse, each having a unique outcome, pattern of disease behavior, and location. CD characteristically causes transmural inflammation that can affect any part of the digestive tract from the mouth to the anus. At the same time, UC is typically limited to the mucosa of the colon. Pathophysiology theories of IBD have evolved over the years with multifactorial etiology, such as genetics, microbiota changes, environmental factors (like cigarette smoking), and immunological factors. 

Generally, the Montreal classification adopts the clinical characterization of IBD [1]. Although reliable, there are limitations in terms of predicting the clinical course of the disease and prognosis. It is essential to predict the probable clinical course accurately. This stratifies patients based on their disease prognosis, enables the selection of more optimal treatments, and reduces the risk of adverse events. Major gastrointestinal organizations worldwide release guidelines for the diagnosis, classification, management, and treatment of IBD; however, there are subtle differences between each of these guidelines. This review focused on highlighting set strengths and divergences in these guidelines to illustrate the best possible management for IBD patients.

## Review

Objective

This commentary aims to review four practice guidelines on inflammatory bowel disease (IBD) and highlight significant similarities and dissimilarities to improve the evidence-based management approach to IBD.

Methodology

We searched PubMed utilizing the keywords: "inflammatory bowel disease," "IBD," "guidelines," "treatment plan," and "diagnosis" in all possible combinations. Four guidelines were identified and used for this review because of their high level of evidence and trusted sources: British Society of Gastroenterology (BSG) consensus guidelines on managing inflammatory bowel disease in adults [2], Canadian Association of Gastroenterology (CAG) Clinical Practice Guideline for the management of inflammatory bowel disease [3], World Gastroenterology Organization (WGO) Global Guidelines on inflammatory bowel disease [4], and American Gastroenterology Association (AGA) clinical practice guideline for the management of inflammatory bowel disease [5]. We extracted information regarding diagnosis, management, and treatment and compared these findings among guidelines reporting the main differences in this review. 

Definition and epidemiology

IBD is a broad term that includes conditions characterized by chronic inflammation of the gastrointestinal tract. IBD includes CD and UC [6]. According to the Centers for Disease Control and Prevention, an estimated 1.3% of United States (US) adults have been diagnosed with IBD in both variants [7]. Reports also showed an increase in incidence from 0.9% in 1999 to 1.3% in 2015 [7-8] and as many as 70,000 diagnosed each year [9].

The prevalence of CD appears to be higher in urban areas than in rural areas and increased in higher socioeconomic classes [4]. If individuals migrate to developed countries before adolescence, those initially belonging to low-incidence populations show a higher incidence of IBD. This is particularly true for the first generation of these families born in a country with a high incidence. In the past 20 years, CD has generally overtaken UC in incidence rates. In developing countries where IBD is emerging, UC is typically more common than CD.

CD characteristically causes transmural inflammation that can affect any part of the digestive tract (from mouth to anus) in a non-continuous pattern. CD can cause complications, such as abscesses, fistulas, and strictures. UC typically only affects the mucosa of the colon. It can extend from the rectum in a proximal and continuous pattern to involve other parts of the colon. Systemic symptoms, such as inflammation of the joints, eyes, and skin, can precede the development of intestinal symptoms [10]. IBD arises in early adulthood but could also start from early childhood. The intestinal manifestations include diarrhea, abdominal pain, and perianal bleeding [11]. 

Pathophysiology

The pathophysiologic theories of IBD have evolved over the years, with emerging research and technological advancements [12]. 

Genetics

In recent studies, genetics have been found to play a more significant role in the development of IBD [13]. A little over 160 gene loci have been identified by internationally collaborative studies associated with CD and are believed to interact closely with environmental and microbial factors.

Environment

Environmental factors, such as smoking, diet consumption, physiological stress, psychological elements, and geography, are considered culprits for IBD. However, smoking has been established to have an inverse association with UC, while it conversely poses a higher risk with CD [14]. Also, vitamin D deficiency has been associated with a higher risk of IBD [15]. Drugs, such as aspirin and nonsteroidal anti-inflammatory drugs, affect the mucosa of the gastrointestinal tract and represent a higher risk for both CD and UC. Antibiotics also act to increase the risk of IBD through their effect on the microbiome. 

Microbial factors

Compared to the defined healthy population, the microbiomes of CD and UC patients deviate from the healthy group, especially in individuals with surgical resection [16]. Studies have compared the microbiome of stool present in inflamed and non-inflamed segments of the intestines. Findings show reduced biodiversity in inflamed areas compared to healthy segments [11]. Recent studies, including studies by Zheng et. al., documented new potential treatment targets based on the findings on the microbiome [17-18]. 

Immunological factors

IBD is secondary to the dysfunction of innate and adaptive immune pathways, which causes an aberrant intestinal inflammatory response in patients with IBD [16].

Nutrition

Some frequently used food components and condiments impact the quality of the intestinal structure and could actuate changes in the intestinal flora, resulting in a pro-inflammatory state favorable for the development of IBD. Foods high in fatty acids and excessive protein intake have been linked to an increased risk of developing IBD. In contrast, increased dietary fiber intake was associated with a decreased risk of developing CD only [19].

Diagnosis

Clinical symptoms, such as weakness, fatigue, long-term diarrhea with abdominal pain, weight variations, and rectal bleeding, may suggest IBD. Diagnostic tests for CD and UC include physical examinations, laboratory tests, and endoscopy [20]. The BSG guidelines recommend ileocolonoscopy, including segmental colonic and ileal biopsies, for IBD disease diagnosis [2]. It is essential to consider that accessing the terminal ileum may not be possible, and small bowel disease could be hard to diagnose through this method. Therefore, if small bowel disease is suspected, a CT enterography following the ileocolonoscopy is recommended. The BSG guidelines also suggest that patients do not require routine upper gastrointestinal endoscopy as part of the diagnosis unless upper gastrointestinal symptoms are present. Where ulcerative colitis is diagnosed by sigmoidoscopy, they recommended a full ileocolonoscopy to delineate disease extent, the severity of inflammation, and to exclude Crohn’s disease (Grade: strong recommendation, very low-quality evidence. Agreement: 100%). 

The diagnosis of IBD, according to the WGO practice guideline, encompasses the patient's history, physical examination, laboratory tests, imaging, and endoscopy [4]. The new guideline by the American Society for Gastrointestinal Endoscopy (ASGE), as documented in the 2017 MAYO clinic report [21], recommended chromoendoscopy as the primary surveillance modality based on its better diagnostic yield than random biopsy approaches [5]. The AGA recommends endoscopic evaluation as soon as the diagnosis of IBD is suspected. With particular emphasis on collecting biopsy samples for pathologic evaluation. The AGA focuses more on the pathological findings than on the appearance of the mucosa at first sight [5] compared to the WGO recommendations [4]. MRI has high levels of sensitivity and specificity for diagnosing CD in the small bowel and may be an alternative to endoscopy. It is also helpful in evaluating perianal disease. It is increasingly being used in pediatric patients and young adults due to the lack of radiation exposure and consequent ability to repeat tests safely. Lastly, the CAG guidelines recommend endoscopy and biopsy; however, they suggest using the Crohn's disease activity index (CDAI) [3]. This is useful to determine the initial activity of the disease and the progression over time. In general, when suggestive symptoms of IBD appear, guidelines suggest an endoscopic biopsy approach.

Location and behavior

The BSG, CAG, WGO, and AGA referenced the Montreal classification of inflammatory bowel disease in their recommendation [1-5]. Classification may help clinicians in patient counseling, disease prognosis, and choice of therapy. The first classification was the Rome classification in 1991; this was later reviewed and changed to the Vienna classification in 1998. Recently, changes have been made to the Vienna version and then the Montreal revision. The Montreal classification takes into consideration the age of diagnosis, location, and behavior [1]. 

Age

The Montreal classification allows early onset of the disease, compared to previous versions [1]. This is important since studies have shown that specific serotypes are more common in the early onset of Crohn's disease. 

Location

Depends on lesion location when IBD is present, including ileal, colonic, ileocolonic, and isolated upper disease. The most significant change in the Montreal revision was adding upper GIT disease [1].

Behavior

This is considered if the disease is stricturing, penetrating, or neither, and separates the perianal condition since recent studies show that patients with this behavior progress differently. The Montreal revision is also the first one to include a subclassification for UC [1]. This new classification is relevant in recent times.

Activity and severity

All consulted guidelines agreed that the determination of disease severity should be based on a combination of symptoms, objective measures of inflammation, and factors that predict an increased risk of complications. This includes CDAI, the Ulcerative Colitis Endoscopic Index of Severity (UCEIS), and the Modified Mayo Endoscopic Score. All three of these scores combine findings in endoscopy, symptoms, and inflammation with a recommendation of Grade: strong recommendation in all guidelines. 

Management and treatment

The management and treatment are determined by the severity of the disease, CD vs. UC, location, comorbidities, physical examination, personal tolerance to the treatment, and access to treatment. 

Diet

The BSG guideline recommends that patients with IBD consider what diets meet their specific requirements, and multidisciplinary management with nutritionists is suggested. In patients where nutritional needs cannot be met, enteral or parenteral nutrition is indicated. Patients should be monitored for dietary parameters like hemoglobin, proteins, vitamins, and electrolytes [2]. The CAG guidelines recommend against the use of enteral nutrition to prevent remissions but with a low level of recommendation [3]. The WGO recommends dietary management using nutritionists and monitoring to prevent malnourishment. Unlike CAG, the WGO guidelines also recommend enteral nutrition to prevent remissions [4]. During disease activity, it is appropriate to decrease the amount of fiber. A low residue diet may decrease the frequency of bowel movements. The AGA recommendations are similar to the BSG guidelines; however, it includes an Anti-inflammatory diet for IBD. This diet incorporates four principles: eating probiotics and prebiotics, avoiding lactose, wheat, and refined sugars; combine vegetables and healthy fats in every meal. Due to a lack of available clinical studies to support the claim [5], this has a low recommendation.

Medical treatment

Oral 5-aminosalicylic acid (5-ASA) is the standard therapy for mild to moderately active IBD. The BSG guideline recommends moderate IBD 5-ASA 2 - 3 g/day with Grade: strong recommendation and high-quality evidence. It also recommends the addition of 5-ASA enemas rather than oral treatment alone. The BSG is the only guideline that suggests enemas. The CAG advises against using oral 5-ASA to induce or maintain complete remission in IBD of any severity [3]. The WGO agrees with the BSG guideline, suggesting the use of 5-ASA in oral or rectal forms. In addition, it recommends combining topical and oral treatment to achieve remission and topical therapy to maintain remission. The AGA also recommends the use of 5-ASA in the oral form for control and initial remission. All consulted guidelines agree that corticosteroids are the second level of treatment in patients with low to moderate IBD, where 5-ASA induction therapy fails, except the CAG; their recommendation is to use corticosteroids as the first option. Besides this difference, the guidelines agree on using topically acting oral corticosteroids like budesonide and beclomethasone dipropionate as an option for patients who do not want to experience the side effects of systemic corticosteroids. Patients with moderate to severe IBD should be treated with oral corticosteroids for at least six to eight weeks. 

Options for Patients Where 5-ASA Failed

Patients on maintenance therapy with high-dose mesalazine, who required two or more corticosteroid courses in the past year, or who become corticosteroid-dependent or refractory, require treatment escalation with thiopurine, anti-TNF therapy, vedolizumab, or tofacitinib. The choice of drug should be determined by clinical factors, patient choice, cost, likely adherence, and local infusion capacity. This is a consistent statement among revised guidelines with Grade: high. 

Surgical management

Surgery is indicated in patients who have become medically resistant, have intolerable side effects, or presents with life-threatening conditions. Surgery should be considered an alternative to medical treatment early in the disease course for short-segment CD limited to the distal ileum [22]. The level of the guidelines' recommendation was "High" and all agreed that IBD patients will need surgery at some point in their lives to relieve symptoms if treatment fails or to correct complications. Surgery performed by a specialist can be curative for the affected areas but is considered an alternative treatment. The suggested procedures are total proctocolectomy (plus permanent ileostomy), ileal pouch-anal anastomosis, and segmental resection (mainly in elderly patients) [23].

Alternative treatments

Treatments like fecal microbial transplantation, marijuana, probiotics, and prebiotics are only mentioned by the BSG and WGO, with a high level of evidence that encourages their use [2, 4]. The CAG and AGA recommend alternative therapies on IBD [3, 5].

Resveratrol

Recent works of literature have shown that resveratrol, found in peanuts, grapes, and red wine, may be helpful [24]. Several studies have expounded on its anti-inflammatory and antioxidant properties to prove its linkage to the improvement of dextran sodium sulfate (DSS)-induced colitis, IL-10−/− chronic colitis in mice, and resveratrol-induced immunosuppressive CD11b+ Gr-1+ cells that express ARG-1, which correlated with reversal of chronic colitis severity [25].

Pomegranate

Studies suggest that the ellagic acid-rich fractions and metabolites of pomegranate urolithin-A provide a protective effect against colitis [26].

Bromelain

Bromelain, which can be gotten from pineapples, has also ameliorated immune-mediated disease properties, including IBD [27]. Further studies on colon biopsies of human IBD patients with bromelain show a decrease in the production of proinflammatory cytokines and chemokines, which suggests a direct effect at effector sites [25, 27-28].

**Table 1:** Comparison of the BSG, CAG, WGO, and AGA Diagnosis, Diet, Treatment, and Other Recommendations AGA: American Gastroenterology Association; anti-TNF: anti-tumor necrosis factor; BSG: British Society of Gastroenterology; CAG: Canadian Association of Gastroenterology; WGO: World Gastroenterology Organization; 5-ASA: 5-aminosalicylic acid

Category	BSG	CAG	WGO	AGA
Diagnosis	Recommends ileocolonoscopy, including segmental colonic and ileal biopsies, if small bowel disease is suspected; follow-up with CT enterography.	Recommends endoscopy and biopsy, using the Crohn’s Disease Activity Index (CDAI) as a predictor	Recommends colonoscopy and sigmoidoscopy for the diagnosis using the Mayo endoscopic score and the Ulcerative Colitis Endoscopic Index of Severity (UCEIS) score	Recommends endoscopic evaluation. Special emphasis on pathology evaluation.
Location and behavior	Used the Montreal classification	Used the Montreal classification	Used the Montreal classification	Used the Montreal classification
Severity Grading	They recommended that disease severity should be based on a combination of symptoms and objective measures of inflammation. They further recommended using Crohn’s Disease Activity Index (CDAI), the Ulcerative Colitis Endoscopic Index of Severity (UCEIS), and the modified Mayo endoscopic score.	They recommended that disease severity should be based on a combination of symptoms and objective measures of inflammation. They further recommended using Crohn’s Disease Activity Index (CDAI), the Ulcerative Colitis Endoscopic Index of Severity (UCEIS), and the modified Mayo endoscopic score.	They recommended that disease severity should be based on a combination of symptoms and objective measures of inflammation. They further recommended using Crohn’s Disease Activity Index (CDAI), the Ulcerative Colitis Endoscopic Index of Severity (UCEIS), and the modified Mayo endoscopic score.	They recommended that disease severity should be based on a combination of symptoms and objective measures of inflammation. They further recommended using Crohn’s Disease Activity Index (CDAI), the Ulcerative Colitis Endoscopic Index of Severity (UCEIS), and the modified Mayo endoscopic score.
Dietary changes	Diet should meet nutritional requirements. Multidisciplinary management with nutritionists. In patients where nutritional requirements cannot be met, enteral or parenteral nutrition is indicated. Patients should be monitored for nutritional parameters, including hemoglobin, proteins, vitamins, and electrolytes.	Recommends against the use of enteral nutrition to prevent remissions, but with a low level of recommendation.	Dietary management with the aid of nutritionists and monitoring to prevent malnourishment. Recommended use of enteral nutrition to prevent remissions.	An anti-inflammatory diet with probiotics and prebiotics, avoid lactose, wheat, and refined sugars; combine vegetables and healthy fats in every meal.
Medical treatment	They recommended 5-ASA. For moderate IBD, they recommended 5-ASA orally, with the addition of 5-ASA enemas. They also recommended corticosteroids used in low to moderate cases only when 5-ASA induction therapy fails. Furthermore, corticosteroids can be used orally or topically. In patients where 5-ASA and corticosteroids failed, treatment escalation with thiopurine, anti-TNF therapy, vedolizumab, or tofacitinib was recommended.	They recommended 5-ASA for any IBD severity and using oral 5-ASA to induce or maintain complete remission. Also, they recommended corticosteroids use from the beginning as first-line therapy. Corticosteroids can also be used orally or topically. In patients where 5-ASA and corticosteroids failed, they recommended that treatment can be escalated with thiopurine, anti-TNF therapy, vedolizumab, or tofacitinib.	They recommended 5-ASA orally, combined with rectal and/or topical treatment. They also recommended corticosteroids use in low to moderate cases only when 5-ASA induction therapy fails. Corticosteroids can also be used orally or topically. In patients where 5-ASA and corticosteroids failed, treatment can be escalated with thiopurine, anti-TNF therapy, vedolizumab, or tofacitinib.	They recommended 5-ASA orally, combined with rectal and/or topical treatment. They also recommended corticosteroid use in low to moderate cases only when 5-ASA induction therapy fails. Corticosteroids can be used orally or topically. In patients where 5-ASA and corticosteroids failed, treatment can be escalated with thiopurine, anti-TNF therapy, vedolizumab, or tofacitinib.
Surgical treatment	In patients that have become medically resistant, have intolerable side effects, or have life-threatening conditions.	In patients that have become medically resistant, have intolerable side effects, or have life-threatening conditions.	In patients that have become medically resistant, have intolerable side effects, or have life-threatening conditions.	In patients that have become medically resistant, have intolerable side effects, or have life-threatening conditions.
Alternative treatment	Fecal microbial transplantation, probiotics, prebiotics, and marijuana	None	Fecal microbial transplantation, probiotics, prebiotics, and marijuana	None

## Conclusions

The management of IBD can be complex and challenging. Thus, guidelines and scores are necessary to make sure the treatment of IBD is universal and comparable. The main differences in the evaluated guidelines were in diagnosis and treatment specifications. The diagnosis of IBD was similar amongst the four guidelines, but some were more specific about limiting the number of interventions necessary to confirm a diagnosis. Their guidelines on treatment had clear suggestions about what was considered ideal. The treatment options were identical; the main differences were on recommended diets and initial therapy in patients with moderate disease. Clinical practice guidelines (CPGs) collaborate and recommend supporting practice evidence from opinion leaders in clinical decision-making. Rather than dictating a one-size-fits-all approach in IBD management, reviewing various guidelines can enhance the cross-pollination of ideas amongst clinicians to improve decision-making. Clearly describing and appraising evidence-based reasoning for scientific recommendations remain driving factors for quality patient care. Despite the effectiveness of CPGs in improving health, the complexities of their formation require constant review to maximize constructive criticisms and explore areas of possible improvements. 

## Appendices

The author and co-authors performed the following roles during the creation of this manuscript:  

Okelue Edwards Okobi - Conceptualization, data curation, formal analysis and interpretation of data, investigation, methodology, project administration, resources, software, validation, visualization, writing - original draft, writing - review and editing, supervision, oversight, and leadership 

Iboro O. Udoete - Conceptualization, formal analysis, investigation, methodology, project administration

Oyinlola O. Fasehun - Resources, validation, visualization, writing - original draft

Tobechukwu Okobi - Project administration, resources, software, validation, visualization, writing - original draft, writing - review and editing.

Endurance O. Evbayekha - Conceptualization, validation, visualization, writing - original draft

Joanna J. Ekabua - Resource and journal review, formal analysis, writing - review and editing

Henry Elukeme - APC drive, methodology, project administration, resources, oversight, and leadership

Imoh L. Ebong - Validation, visualization, writing - original draft, writing - review and editing, and leadership.

Olamide O. Ajayi - Conceptualization, validation, visualization, writing - original draft, visualization, writing - initial draft,

Iyanu V. Olateju - Visualization, writing - original draft, resources, software, validation

Anthonette Taiwo - Investigation, methodology, project administration, writing - original draft, writing - review and editing

Ifeoma C. Anaya - Conceptualization, validation, visualization, sriting - original draft.

Janet A. Omole - Methodology, project administration, resources, data curation

Mireille B. Nkongho - Writing of the article, reviewing and/or revising the text and/or figures

Ugochi Ojinnaka - Writing of the article, reviewing and/or revising the text, visualization

Abimbola O. Ajibowo - Project administration, drafting or revising the article, resources

Omosefe E. Ogbeifun - Validation, visualization, drafting, or revising the article

Osemwegie O. Ugbo - Drafting or revising the article, writing - review and editing

Ovie Okorare - Editing, writing, and review of the original draft

Zainab Akinsola - Editing, concept rephrasing, and maintenance of research data integrity

Rahman A. Olusoji - Literature search, writing, and editing

Ijeoma O. Amanze - Preparation, typing, literature search, and editing

Jane N. Nwafor - Formal analysis of methodology, writing, and drafting of conclusion

Nnenna A. Ukoha - Drafting or revising the article, writing - review and editing

Thomas A. Elimihele - Project administration, writing - original draft, writing - review and editing
